# Evaluation of the antiviral activity of orlistat (tetrahydrolipstatin) against dengue virus, Japanese encephalitis virus, Zika virus and chikungunya virus

**DOI:** 10.1038/s41598-020-58468-8

**Published:** 2020-01-30

**Authors:** Atitaya Hitakarun, Sarawut Khongwichit, Nitwara Wikan, Sittiruk Roytrakul, Sutee Yoksan, Supoth Rajakam, Andrew D. Davidson, Duncan R. Smith

**Affiliations:** 1 Institute of Molecular Biosciences, Mahidol University, Salaya, 73170 Thailand; 2 National Center for Genetic Engineering and Biotechnology (BIOTEC), National Science and Technology Development Agency, Pathum Thani, 12120 Thailand; 3 School of Cellular and Molecular Medicine, University of Bristol, Bristol, BS8 1TD United Kingdom

**Keywords:** Virology, Drug discovery

## Abstract

Many mosquito transmitted viruses of the genera *Alphavirus* and *Flavivirus* are human pathogens of significant concern, and there is currently no specific antiviral for any member of these two genera. This study sought to investigate the broad utility of orlistat (tetrahydrolipstatin) in reducing virus infection for several mosquito borne viruses including flaviviruses (dengue virus (DENV; nine isolates analyzed), Japanese encephalitis virus (JEV; one isolate analyzed) and Zika virus (ZIKV; 2 isolates analyzed)) as well as an alphavirus (chikungunya virus; CHIKV; 2 isolates analyzed). Three different treatment regimens were evaluated, namely pre-treatment (only), post-treatment (only) and pre- and post-treatment, and three factors were evaluated, namely level of infection, virus titer and genome copy number. Results showed that all three treatment modalities were able to significantly reduce virus titer for all viruses investigated, with the exception of three isolates of DENV in the pre-treatment only regimen. Pre- and post-treatment was more effective in reducing the level of infection and genome copy number of all viruses investigated than either pre-treatment or post-treatment alone. Collectively, these results suggest orlistat has potential as a broad-spectrum agent against multiple mosquito transmitted viruses.

## Introduction

Mosquito transmitted viruses are a significant public health problem in many tropical and sub-tropical countries, and some of the most significant human pathogens belong to the genera *Flavivirus* and *Alphavirus*^1^. The genus *Flavivirus* consists of 53 virus species^2^ of which more than half are transmitted by mosquitoes and the majority of these have the potential to infect humans^3^. Medically important mosquito transmitted viruses in the genus *Flavivirus* include dengue virus (DENV), Japanese encephalitis virus (JEV), Zika virus (ZIKV) and yellow fever virus (YFV). The genus *Alphavirus* consists of 31 virus species^2^ the majority of which are spread by mosquitoes, and medically important alphaviruses include chikungunya virus (CHIKV), Ross River virus, Semliki Forest Virus and Sindbis virus^4^.

Viruses in the genera *Flavivirus* and *Alphavirus* have a number of similarities. Viruses in both genera are classified in group IV in the Baltimore classification system^5^ as they possess a positive sense single stranded RNA genome. The genome sizes are approximately equivalent (flaviviruses approximately 9.2–11 kb^6^, alphaviruses approximately 9.7–12 kb^7^, but while the ten flavivirus proteins (capsid (C), pre-membrane (prM), envelope (E), NS1, NS2A, NS2B, NS3, NS4A, NS4B and NS5) are encoded by a single open reading frame, the nine alphavirus proteins are encoded by two open reading frames, the first of which encodes the non-structural proteins (nsP1, nsP2, nsP3 and nsP4), while the second open reading frame encodes the structural proteins (C, E1, E2, E3), as well as a protein (6 K) of uncertain function^4^. Viruses in both genera encode a protein with RNA-dependent RNA polymerase (RdRP) activity that undertakes genome replication. Both the flavivirus RdRP activity containing protein (NS5) and the alphavirus RdRP activity containing protein (nsP4) lack proof reading activity, and thus replication is error prone, leading to high rates of mutation^8^. The high rate of mutation leads to significant diversity as well as the possibility of sudden emergence of variants with enhanced pathogenic potential. This was typified by the rapid spread of CHIKV between 2004 and 2010 to many countries around the Indian Ocean^9^ that was believed to be driven by a mutation enhancing transmission in *Aedes albopictus* mosquitoes^10^. Some evidence has suggested that the recent spread of ZIKV (reviewed in^11^) may be driven by mutations similarly enhancing virus transmission^12^.

Viruses in both genera are enveloped viruses. During virus replication the newly synthesized genomic RNA is packaged together with the capsid protein which is then enveloped in a host derived lipid bilayer in which the viral structural envelope proteins are embedded^4^. As a result of this step, host derived lipid makes up an estimated 17% of the flavivirus and 30% of the alphavirus virion total weight^13^.

Several studies, particularly those undertaken with flaviviruses, have clearly shown the requirement of host cell lipid metabolism for viral replication and assembly, and currently it is believed that flaviviruses re-model host cell metabolism to facilitate their own replication (reviewed in^14^). Lipid remodelling has been proposed to facilitate increased β-oxidation to provide energy for replication^15^, as well as changing membrane fluidity to allow correct assembly of the virion^16^. In addition, lipid droplets have been proposed to be essential for correct encapsulation of the nucleocapsid^17^. The possibly multi-step requirement for host cell lipids during viral replication suggests that these processes are attractive targets for anti-viral drug development.

Orlistat (tetrahydrolipstatin), a US Food and Drug Administration (FDA) approved drug, inhibits the thioesterase domain of fatty acid synthase (FASN) a key enzyme responsible for the *de novo* synthesis of long chain fatty acids^18^. This drug has been used as a weight loss medication that acts through the inhibition of pancreatic lipases, resulting in decreased absorption of fats from diet, and to increase the secretion of fats in stool^19^. Because high levels of fatty acid from lipid biosynthesis can promote the rapid growth and division of cancer cells, orlistat has additionally been evaluated for the therapy of a number of cancers including endometrial, prostate, breast, and colorectal cancers^20–25^. Orlistat has also been evaluated as an antiviral agent, and previous studies have evaluated the usefulness of orlistat as an antiviral agent for coxsackievirus B3 and varicella-zoster virus^26^. In our previous study we showed the utility of orlistat as an antiviral agent against DENV^27^, however that study only investigated DENV serotypes 2 and 4. As noted earlier, flaviviruses have no RdRP proof reading activity, and as such show significant variation. DENV is no exception, and in addition to the existence of four DENV serotypes^28^, these are further separated into multiple lineages and strains. How such diversity can affect the antiviral activity of drugs against a particular virus remains unclear. This study sought to determine the antiviral activity of orlistat against a number of different DENV isolates, as well as JEV, ZIKV and CHIKV. The HEK293T/17 cell line^29^ was selected for this study as it has been shown to be highly susceptible to a number of DENV isolates. As studies have shown that oral administration of orlistat results in very low levels of systemic absorption^30,31^, and that levels can decrease to undetectable in a few hours, three different treatment regimens were investigated, namely a short pulse pre-treatment, as well as post-infection treatment and a combined pre-and post-treatment.

## Materials and Methods

### Cell lines and cell culture conditions

C6/36 cells (ATCC^®^CRL-1660^™^), were cultured at 28 °C in minimum essential medium (MEM, GIBCO, Invitrogen, Grand Island, NY) supplemented with 10% fetal bovine serum (FBS) and 100 units/ml of penicillin/streptomycin (GIBCO, Invitrogen). LLC-MK_2_ (ATCC^®^CCL-7^™^) and Vero (ATCC^®^CCL-81^™^) cells were cultured at 37 °C with 5% CO_2_ in Dulbecco’s modified Eagle’s medium (DMEM, GIBCO, Invitrogen) supplemented with 5% FBS and 100 units/ml of penicillin/streptomycin. HEK293T/17 cells (ATCC^®^ CRL-11268^™^) were cultured at 37 °C with 5% CO_2_ and maintained in DMEM supplemented with 10% FBS and 100 units/ml of penicillin/streptomycin.

### Viruses and infections

DENV (Family *Flaviviridae*, Genus *Flaviviru*s, Species *Dengue virus*) isolates consisted of representatives of the four DENV serotypes including five laboratory adapted high passage isolates, namely DENV 1 strain 16007 (DENV 1LAB), DENV 1-Hawaii, DENV 2 strain 16681 (DENV 2LAB), DENV 3 strain 16562 (DENV 3LAB), and DENV 4 strain 1036 (DENV 4LAB), four low passage strains originally isolated from dengue fever patients, namely SS12/61 (DENV 1DF), SS12/62 (DENV 2DF), SS12/64 (DENV 3DF) and SS12/66 (DENV 4DF), four low passage strains originally from dengue haemorrhagic fever patients namely SS12/60 (DENV 1DHF), SS12/63 (DENV 2DHF), SS12/65 (DENV 3DHF), and SS12/67 (DENV 4DHF), and five low passage strains originally isolated from undifferentiated fever patients SS11/1666 (DENV 1/1666), SS15/1113 (DENV 3/1113), SS14/146 (DENV 4/146), SS14/163 (DENV 4/163), and SS11/1373 (DENV 4/1373). ZIKV (Family *Flaviviridae*, Genus *Flaviviru*s, Species *Zika virus*) isolates included an Asian lineage virus (SV0010/15) kindly provided by the Armed Forces Research Institution of Medical Sciences (AFRIMS) and the Development of Disease Control, Ministry of Public Health, Thailand and the African lineage virus MR766. The JEV isolate (Family *Flaviviridae*, Genus *Flaviviru*s, Species *Japanese encephalitis virus*) was the Beijing 1 (BJ1) strain. CHIKV isolates (Family *Togaviridae*, Genus *Alphavirus*, Species *Chikungunya virus*) consisted of two isolates of the East Central and South African (ECSA) lineage ECSA 226 V and ECSA A226 as previously described^32^. A summary of the viruses used in this study is shown in Supplemental Table 1. All work with live viruses was undertaken in a BSL2 laboratory after approval by the Institutional (Mahidol University) Biosafety Committee.

All viruses were propagated in confluent C6/36 (for the flaviviruses) or Vero (for the alphavirus) cells pregrown in 175 cm^2^ tissue culture flasks. Cells were infected with each virus at a multiplicity of infection (MOI) of 1 in MEM medium (C6/36) or DMEM (Vero) for 2 h at 28 °C (C6/36) or 37 °C (Vero) with constant agitation, following which complete medium was added and infected cells were further incubated under standard conditions. At the time of highest cytopathic effects, the culture medium was centrifuged to remove cell debris and supplemented with FBS to a final concentration of 20% (v/v). Viruses were kept at −80 °C until use.

### Standard plaque assay

To determine the viral titer or viral production, LLC-MK_2_ or Vero cells were plated at a density that allowed confluence to be reached within 24 h. Monolayers were infected with viruses 10-fold serially diluted in 1 × M-199E medium (GIBCO, Invitrogen) for 2 h at 37 °C with constant agitation following which the infected cells were overlaid with 0.8% Seakem Le agarose (Cambrex, East Rutherford, NJ) coupled with 2X nutrient solution (Earle’s Balanced Salts supplemented with 0.33% (w/v) yeast extract, 0.165% lactalbumin hydrolysate, and 3% FBS for DENV, JEV, and CHIKV or 1.2% Methyl cellulose (Merck KGaA) in 2X DMEM supplemented with 2% FBS for ZIKV. The plates were incubated at 37 °C, 5% CO_2_ for 3 (CHIKV), or 6 (DENV, and JEV) days before a second overlay with 0.8% Seakem Le agarose mixed with nutrient containing neutral red and incubated overnight before plaque counting. For ZIKV after incubation at 37 °C, 5% CO_2_ for 7 days plates were fixed with 3.7% (v/v) formaldehyde (Merck KGaA) in 1X Phosphate Buffer Saline (PBS) for 2 h, and stained with 1% crystal violet dye prior to plaque counting. The plaques were counted and viral titer was determined in terms of plaque forming unit/ml (pfu/ml).

### Flow cytometry

Mock infected or virus infected cells were collected at appropriate time points post-infection and washed twice with 1X PBS. Cells were blocked with 10% normal goat serum (Gibco BRL) for 30 min on ice before washing twice with 1% bovine serum albumin (BSA) (Carpicorn Scientific GMbH, Germany) in 1X PBS following which cells were fixed with 4% paraformaldehyde (Merck KGaA) in 1X PBS in the dark at room temperature for 20 min following by washing with 1% BSA in 1X PBS. Cells were permeabilized with 0.2% Triton X-100 (OmniPur, Merck KGaA) in 1% BSA in 1X PBS and washed twice with 1% BSA in 1X PBS. Cells were incubated overnight at 4 °C with an appropriate primary antibody, namely a 1:150 dilution of pan-specific mouse monoclonal anti-dengue E protein antibody (HB114^33^), for the detection of DENV infected cells, and a combination between a 1:150 dilution of HB114 with a 1:3 dilution of pan specific mouse monoclonal anti-flavivirus antibody (HB112) for ZIKV and JEV, or a 1:200 dilution of a mouse monoclonal anti-alphavirus antibody (sc-58088, Santa Cruz Biotechnology, Santa Cruz, CA) for CHIKV infected cells. After two washes with 1% BSA, cells were incubated with a 1:40 dilution of a FITC conjugated goat-anti mouse IgG antibody (KPL, Gaitherburg, MD) in the dark at room temperature for 1 h. Finally, cells were washed twice and resuspended in 1X PBS and analyzed on a flow cytometer (BD, FACSCalibur) using the CELLQuest^TM^ software (BD Biosciences). All experiments were undertaken independently in triplicate.

### Determination of cytotoxicity of orlistat

Cytotoxicity of orlistat (Merck KGaA) was evaluated in HEK293T/17 cells at 36 h with pre-, post-, or pre- and post-combined treatments using a Trypan Blue viability testing protocol. Dilutions of cell suspensions were mixed 1:1 with 0.4% Trypan Blue prior to counting live cells using a hemocytometer under a microscope. Negative controls (only DMEM and vehicle DMSO control of each concentration) and positive controls (milli-Q water) were undertaken in parallel. In addition, cell morphology was evaluated under an inverted light microscope.

### Evaluation of antiviral activity of orlistat

HEK293T/17 cells were seeded into 6-well plates at a density that allowed 80–90% confluence to be reached within 24 h at 37 °C with 5% CO_2_. Cells were then pre-treated, post-treated, or pre- and post-combination treated with 100 μM of orlistat. For pre-treatment cells were incubated with media containing orlistat or DMSO vehicle control for one hour following which cells were mock infected or infected with the appropriate virus at MOI 5 in DMEM medium for 2 h after which the medium was removed. After infection cells were washed once with PBS before the addition of complete medium (DMEM supplemented with FBS and antibiotics) without orlistat for pre-treatment only, or with orlistat for post- and pre- and post-combined treatments and incubated under standard conditions. Cells were incubated for 36 h post infection (h.p.i.) before cells and/or culture medium was collected for further analysis. All experiments were undertaken independently in triplicate.

### Quantitative real time RT-PCR (qRT-PCR)

Total RNA was extracted from culture medium using a 1:1 proportion of culture medium and TRI reagent solution (Molecular Research Center, Inc., Cincinnati, OH) according to the manufacturers’ protocol. The final RNA pellet was resuspended with DEPC-treated water. The concentration of the extracted RNA was measured using a Nanodrop ND-1000 UV-Vis spectrophotometer and subsequently adjusted to the final concentration using DEPC-treated water.

cDNA was prepared from the adjusted total RNA using Thermo Scientific RevertAid Reverse Transcriptase (Thermo Fisher Scientific, Waltham, MA) and random hexamer primers (Thermo Fisher Scientific). The cDNA was diluted and used as a template for qRT-PCR. Quantitative real time PCR was performed based on the SYBR technique using KAPA SYBR® FAST qPCR Kit 2X Master MIX (Kapa Biosystems Inc, Woburn, MA.) in a Mastercycler^®^ ep realplex real-time PCR machine using specific primers (Supplemental Table 2). The PCR amplifications were performed at 95 °C for 3 min, followed by 40 cycles of 95 °C for 10 sec, 60 °C for 30 sec, and 72 °C for 20 sec.

The PCR product of each standard was purified by FavorPrep GEL/PCR Purification Mini Kit (Favogen BioTech, Taiwan) and subsequently diluted to various copy numbers following calculation using the Thermo Scientific Web Tool (https://www.thermofisher.com/th/en/home/brands/thermo-scientific/molecular-biology/molecular-biology-learning-center/molecular-biology-resource-library/thermo-scientific-web-tools/dna-copy-number-calculator.html). Determination of genome copy number for each virus was calculated as copies/ml based on genome length and concentration and comparison with a virus specific standard curve generated using ten-fold serial dilution (10^0^ to 10^10^) of the viral genome.

### Western blot analysis

Approximately 60 μg of protein per sample was heated to 100 °C for 5 min and then separated by electrophoresis through a 12% sodium dodecyl sulfate polyacrylamide gel (SDS-PAGE). The proteins were subsequently transferred to nitrocellulose membranes (Whatman GmbH, Germany) using a Trans-Blot electrophoretic transfer cell (Bio-Rad Laboratories, Richmond, CA). After protein transfer the membranes were blocked with 5% skimmed milk (w/v) in Tris-buffered saline (TBS-T) containing 0.05% Tween 20 at room temperature for 2 h or at 4 °C overnight after which membranes were washed three times with TBS-T for 5 min each time. Detection of both viral and host proteins was performed by incubating the membrane at 4 °C overnight with an appropriate primary antibody, namely a 1:500 dilution of pan specific mouse monoclonal anti-flavivirus antibody (HB112), a 1:2000 dilution of a rabbit anti-DENV 2 NS1 protein polyclonal antibody (PA5-27885, Thermo Fisher Scientific), a 1:1000 dilution of a rabbit anti anti-DENV 2 NS3 polyclonal antibody (GTX124252, GeneTex), a 1:1000 dilution of a rabbit anti-DENV 2 NS5 polyclonal antibody (PA5-27888, Thermo Fisher Scientific) or a 1:2000 dilution of an anti-GAPDH mouse monoclonal antibody (sc-32233; Santa Cruz Biotechnology Inc.). After incubation, membranes were washed three times with TBS-T and incubated with an appropriate secondary antibody conjugated with HRP at room temperature for 1 h. The signals were developed using the Amersham ECL plus Western Blotting Detection Reagents (GE Healthcare) and immediately detected using the ChemiDoc XRS^+^ system with Image Lab^TM^ software (BioRad, Hercules, CA).

### Statistical analysis

All data were analysed using the GraphPad Prism software program version 7.0a (GraphPad Software Inc., San Diego, CA) and results are presented as mean ± the standard error of the mean (SEM). The statistical analysis was analysed using independent sample *t*-test (and nonparametric tests) in GraphPad Prism software program. Data was considered as statistically significant at a *p* value < 0.05.

## Results

### Orlistat cytotoxicity assessment

Although we have previously evaluated the cytotoxicity of orlistat in HEK293T/17 cells^27^ that was for a single treatment regimen (pre- and post-infection treatment) and so the cytotoxicity was re-determined for the three treatment profiles to be used in this study (pre-infection treatment only, post-infection treatment only and treatment both pre- and post-infection). The 50% cytotoxic concentrations (CC_50_) of orlistat in HEK293T/17 cells at 36 h were determined to be 2715.25 μM (pre-treatment only), 517.55 μM (post-treatment only) and 402.43 μM (pre-and post-treatment) (Supplemental Fig. 1). The figure for the combined pre- and post- treatment was in relatively good agreement with our previous study^27^, given the methodological differences. Some changes in cell morphology were observed at 36 h after treatment with 200 μM of orlistat in both post-, and pre- and post-combined treatment (Supplemental Figs. 2–4). All screening was therefore undertaken at a concentration of 100 μM of orlistat, consistent with our previous study^27^.

### Optimization of infection

**Figure 1:**
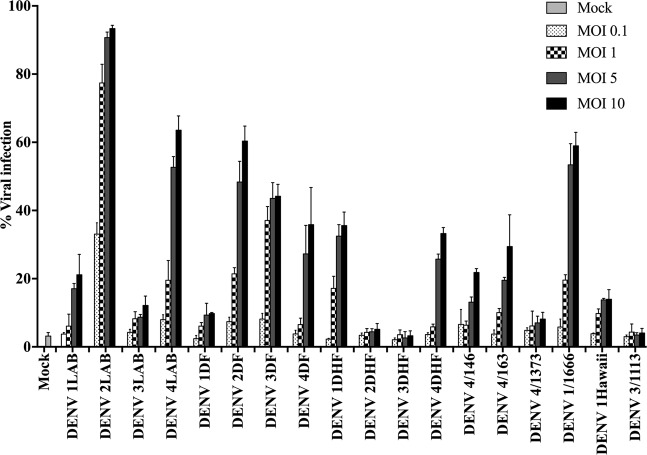
Evaluation of DENV infection of HEK293T/17 cells. HEK293/17 cells were mock infected or infected with one of 18 DENVs namely DENV 1LAB, DENV 1-Hawaii, DENV 2LAB, DENV 3LAB, DENV 4LAB, DENV 1DF, DENV 2DF, DENV 3DF, DENV 4DF, DENV 1DHF, DENV 2DHF, DENV 3DHF, DENV 4DHF, DENV 1/1666, DENV 3/1113, DENV 4/146, DENV 4/163, and DENV 4/1373 at MOIs ranging from 0.1 to 10 and the level of infection was determined at 36 h.p.i by flow cytometry. All experiments were undertaken independently in triplicate.

A total of 18 DENV isolates were obtained, stock virus prepared and titered, and the identity of the viruses confirmed by DNA sequencing using consensus primers D1 and D2 as developed by Lanciotti and colleagues^34^. Twelve of these viruses have been previously described^35^, and the viruses are summarized in Supplemental Table 1. HEK293T/17 cells were infected separately with all 18 DENVs at a range of MOIs, and the degree of infection determined at 24 and 36 h.p.i by flow cytometry. The results showed a wide range of infectivity at 24 h.p.i (Supplemental Fig. 5) ranging from approximately 85% (DENV 2LAB) at MOI 10 to effectively undetectable (e.g. DENV 3DHF at all MOI). Only one virus (DENV 2LAB) showed infection levels above 40% for all MOI investigated. By 36 h.p.i infection, levels had increased for all viruses, and nine DENV showed infection levels of 30% or above (Fig. 1), and these viruses (DENV 1DHF, DENV 1/1666, DENV 2LAB, DENV 2DF, DENV 3DF, DENV 4LAB, DENV 4DF, DENV 4DHF, DENV 4/163) were selected for further evaluation.

### Effect of orlistat on viral infection and production

**Figure 2:**
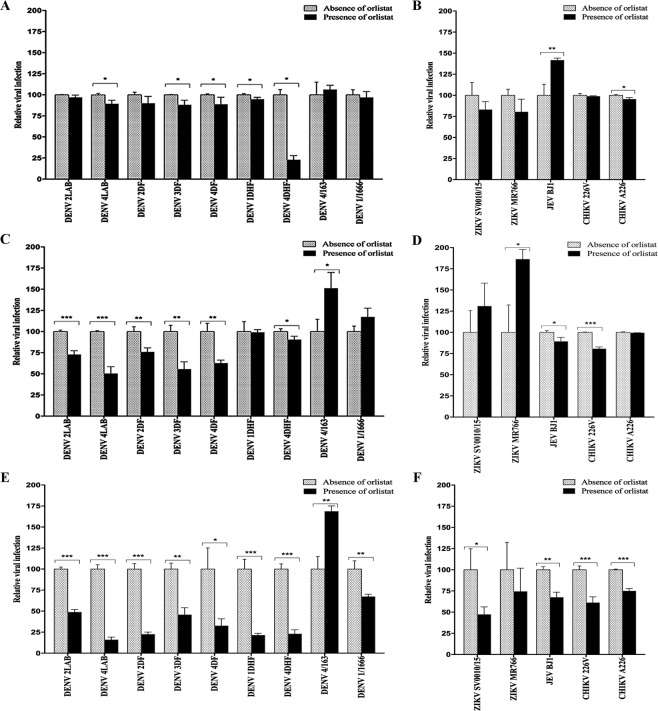
Effect of orlistat treatment on infection. HEK293T/17 cells were treated with either DMSO vehicle control or 100 μM orlistat for (**A**,**B**) 1 h pre-infection only, (**C**,**D**) post-infection only, or (**E**,**F**) pre- and post-infection and were infected at MOI of 5 with (**A**,**C** and **E**) nine DENVs representing the four serotypes of DENV (DENV 1DHF, DENV 1/1666, DENV 2LAB, DENV 2DF, DENV 3DF, DENV 4LAB, DENV 4DF, DENV 4DHF, DENV 4/163), and (**B**,**D** and **F**) two isolates of ZIKV (SV0010/15 and MR766), one isolate of JEV (BJ1), and two isolates of CHIKV (ECSA 226 V and ECSA A226). At 36 h.p.i. the percentage infection was determined by flow cytometry. Experiments were undertaken independently in triplicate. Bar graphs show mean +/− SD (*p value < 0.05, **p value < 0.01, and ***p value < 0.001).

The effect of orlistat on a range of viruses, including nine isolates of DENV as well as two isolates of ZIKV, one isolate of JEV and two isolates of CHIKV (Supplemental Table 1) was investigated using three different treatment regimens. For the pre-treatment procedure, HEK293T/17 cells were incubated with either 100 μM of orlistat or DMSO vehicle control (0.26% DMSO) for 1 h and then infected with the selected viruses at an MOI of 5 for 2 h in the absence of the drug. After removal of the viruses by washing, the cells were cultured in complete media without orlistat under standard conditions for 36 h. For the post-treatment regimen, HEK293T/17 cells were infected with the selected viruses at an MOI of 5 for 2 h in the absence of the drug, after which the viruses were removed by washing and the cells cultured in complete medium including orlistat or vehicle control for 36 h. Finally for pre- and post- treatment, cells were treated for 1 h with either the drug or vehicle control, infected with the selected viruses in the absence of the drug and after removal of the viruses by washing, the cells were incubated in complete medium with the drug or vehicle control as appropriate for 36 h after which the level of viral infection was determined by flow cytometry. The pre-treatment regime resulted in a significant reduction in the level of infected cells for five of the nine DENV isolates examined (Fig. 2A), and no significant reduction of the levels of cells infected with ZIKV (Fig. 2B). A small but significant reduction was seen with one of the two CHIKV isolates (Fig. 2B). Surprisingly, pre-treatment of cells with orlistat significantly increased levels of JEV infection (Fig. 2B). The post-treatment regime resulted in a significant reduction in the level of infected cells for six DENV isolates, JEV, and one of the two CHIKV isolates (Fig. 2C,D), but a significant increase in the level of infection was seen for the DENV 4/163 and ZIKV MR766 isolates (Fig. 2C,D). The combined pre- and post-treatment regime resulted in a significant reduction in levels of infection for eight DENV isolates, ZIKV SV0010/15, JEV, and both isolates of CHIKV. Notably, DENV 4/163 still showed increased levels of infection. The non-normalized data for the ZIKV, JEV and CHIKV cell infection experiments is shown in Supplemental Fig. 6.

**Figure 3:**
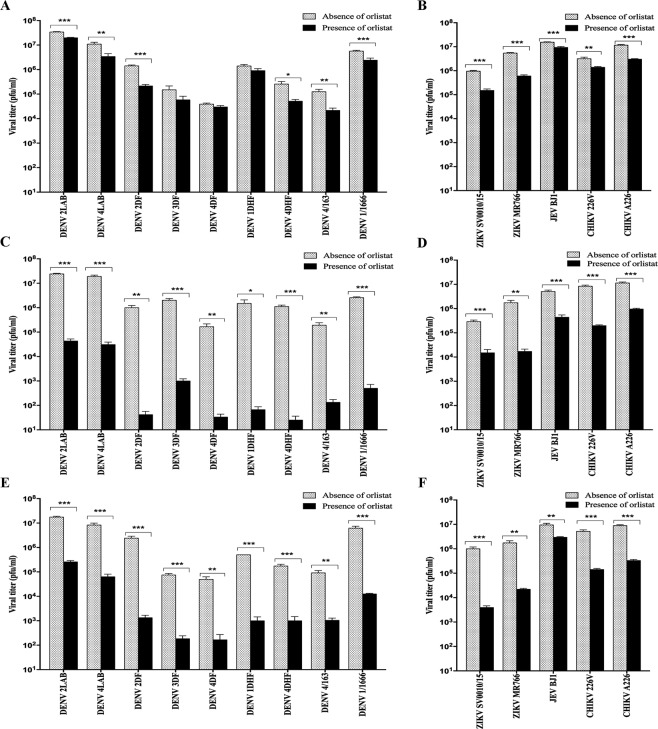
Effect of orlistat treatment on virus titer. HEK293T/17 cells were treated with DMSO vehicle control or 100 μM orlistat for (**A**,**B**) 1 h pre-infection only, (**C**,**D**) post-infection only, or (**E**,**F**) pre- and post-infection and were infected at MOI of 5 with (**A**,**C** and **E**) nine DENVs representing the four serotypes of DENV (DENV 1DHF, DENV 1/1666, DENV 2LAB, DENV 2DF, DENV 3DF, DENV 4LAB, DENV 4DF, DENV 4DHF, DENV 4/163), and (**B**,**D** and **F**) two isolates of ZIKV (SV0010/15 and MR766), one isolate of JEV (BJ1), and two isolates of CHIKV (ECSA 226 V and ECSA A226). At 36 h.p.i. viral titers in the supernatants were determined by standard plaque assay. Experiments were undertaken independently in triplicate with duplicate plaque assay. Bar graphs show mean +/− SD (*p value < 0.05, **p value < 0.01, and ***p value < 0.001).

In addition to determining the level of infection, the virus titer in the cell supernatants was determined by standard plaque assay, and results showed somewhat more uniform effects. Pre-treatment resulted in a small (<1 log) but significant reduction in virus titer for six of the nine DENV isolates examined (Fig. 3A), as well as for both ZIKV (SV0010/15 and MR766) and CHIKV (CHIKV 226 V and CHIKVA226) isolates (Fig. 3B). In contrast to the analysis of JEV infection levels which showed increased levels of infection (Fig. 2B), a significant reduction in virus titer was observed for JEV (Fig. 3B). The reduction in ZIKV virus titer was on the order of 1 log. Given the discordant results for JEV, in which levels of infection were increased, while virus titer was decreased, the experiment was repeated, and this the time level of infection and virus titer were determined at 12 and 36 h.p.i. The results (Supplemental Fig. 7) confirmed the previous experiment and showed that at both time points, the levels of infection were higher in the treated cells, but the virus titer in the supernatant was lower. When the cells were treated with orlistat either post-treatment or pre- and post-treatment, a significant effect on viral production was observed for all viruses investigated, with reductions in the order of 2–4 log for all nine DENV isolates (Fig. 3C,E), 1–2 log for ZIKV, 1 log for JEV, and 1–2 log for CHIKV (Fig. 3D,F).

### Effect of orlistat on genome copy number

**Figure 4:**
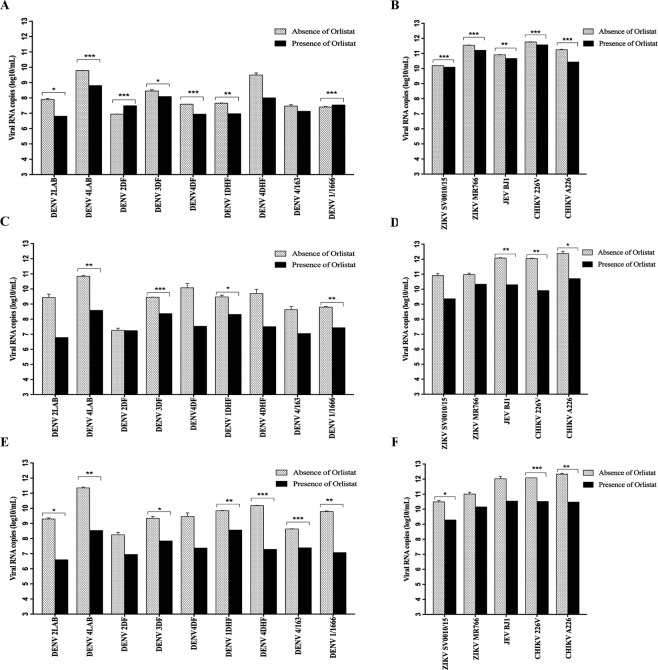
Effect of orlistat treatment on genome copy number. HEK293T/17 cells were treated with either DMSO vehicle control or 100 μM orlistat for (**A**,**B**) 1 h pre-infection only, (**C**,**D**) post-infection only, or (**E**,**F**) pre- and post-infection and were infected at MOI of 5 with (**A**,**C** and **E**) nine DENVs representing the four serotypes of DENV (DENV 1DHF, DENV 1/1666, DENV 2LAB, DENV 2DF, DENV 3DF, DENV 4LAB, DENV 4DF, DENV 4DHF, DENV 4/163), and (**B**,**D** and **F**) two isolates of ZIKV (SV0010/15 and MR766), one isolate of JEV (BJ1), and two isolates of CHIKV (ECSA 226 V and ECSA A226). At 36 h.p.i. the supernatants were harvested and RNA extracted. Quantification of viral RNA was undertaken by qRT-PCR. Bar graphs show mean +/− SD (*p value < 0.05, **p value < 0.01, and ***p value < 0.001).

To determine the effect of orlistat on viral RNA copy number, supernatants from cells either infected using the different orlistat treatment regimes or not treated were evalauted by qRT- PCR. This experiment was undertaken independently from the previous analysis. The results were analyzed in comparison with standard curves generated by ten-fold serial dilutions of the appropriate virus. When cells were pre-treated with orlistat prior to infection, there was a significant reduction in viral genome copy number for five out of nine DENV isolates (Fig. 4A). The four exceptions were the DENV 4DHF and DENV 4/163 isolates in which the reduction in genome copy number did not reach significance, while DENV 2DF and DENV 1/1666 showed a significant increase in genome copy number (Fig. 4A). For the other viruses (Fig. 4B) a significant reduction in genome copy number occurred for both strains of ZIKV, JEV and two isolates of CHIKV (Fig. 4B). In contrast, longer-term treatment of the cells with orlistat (post-treatment or pre- and post-treatment) resulted in a significant reduction in viral copy number for four (post-treatment, Fig. 4C) or seven (pre- and post-treatment, Fig. 4E) out of nine DENV isolates. Notably however, the remaining DENV isolates showed reduced genome copy numbers albeit to levels that did not reach statistical significance. One of the two ZIKV isolates showed a reduction in genome copy number as a consequence of orlistat pre- and post-combined treatment (Fig. 4F). The JEV isolate showed a reduction in genome copy number that only reached significant levels for the post-treatment regime, whilst both isolates of CHIKV exhibited a significant reduction in genome copy number for both longer-term treatments (Fig. 4D,F). A summary of the effects of orlistat on the level of virus infection, virus titer and genome copy number under the different treatment regimens is shown in Table 1.

**Table 1:** Summary of effect of orlistat treatment under different treatment regimens on virus replication parameters.

Virus	Level of infection			Virus titer			Genome copy number		
	Pre-	Post-	Pre- and Post-	Pre-	Post-	Pre- and Post-	Pre-	Post-	Pre- and Post-
DENV 1DHF	↓	—^a^	↓	—^a^	↓	↓	↓	↓	↓
DENV 1/1666	—^a^	—^a^	↓	↓	↓	↓	↑	↓	↓
DENV 2LAB	—^a^	↓	↓	↓	↓	↓	↓	—^a^	↓
DENV 2DF	—^a^	↓	↓	↓	↓	↓	↑	—^a^	—^a^
DENV 3DF	↓	↓	↓	—^a^	↓	↓	↓	↓	↓
DENV 4LAB	↓	↓	↓	↓	↓	↓	↓	↓	↓
DENV 4DF	↓	↓	↓	—^a^	↓	↓	↓	—^a^	—^a^
DENV 4DHF	↓	↓	↓	↓	↓	↓	—^a^	—^a^	↓
DENV 4/163	—^a^	↑	↑	↓	↓	↓	—^a^	—^a^	↓
ZIKV SV0010/15	—^a^	—^a^	↓	↓	↓	↓	↓	—^a^	↓
ZIKV-MR766	—^a^	↑	—^a^	↓	↓	↓	↓	—^a^	—^a^
JEV BJ1	↑	↓	↓	↓	↓	↓	↓	↓	—^a^
CHIKV ECSA 226 V	—^a^	↓	↓	↓	↓	↓	↓	↓	↓
CHIKV ECSA A226	↓	^a^	↓	↓	↓	↓	↓	↓	↓

^a^Symbol “ —” indicates no significant change (p value > 0.05).

### Effect of orlistat on viral protein expression

**Figure 5:**
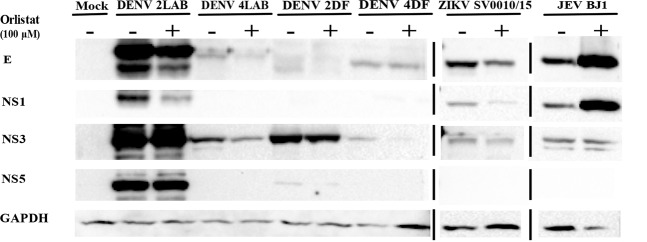
Effect of orlistat pre-treatment on viral protein expression. HEK293T/17 cells were pre-treated with a DMSO vehicle control or 100 μM orlistat and were subsequently infected at MOI of 5 with DENV (DENV 2LAB, DENV 4LAB, DENV 2DF and DENV 4DF), ZIKV (SV0010/15) or JEV (BJ1). At 36 h.p.i. the level of the E, NS1, NS3, and NS5 proteins were determined by western blotting. All membranes were stripped and re-probed with GAPDH as a loading control. Consecutive probings are separated by white spaces, non-contiguous bands are separated by black lines.

We have previously shown the effect of orlistat on DENV protein expression under a combined pre- and post-infection treatment protocol^27^. To determine the effect of orlistat on viral protein expression under the minimal treatment regime (pre-treatment only), cells were not pre-treated, or pre-treated with orlistat for 1 h before infection with DENV (DENV 2LAB, DENV 4LAB, DENV 2DF and DENV 4DF), ZIKV (SV0010/15) or JEV (BJ1). This experiment was undertaken independently from the previous analysis. At 36 h.p.i. proteins were extracted and subjected to western blot analysis to detect the viral E, NS1, NS3, and NS5 proteins. Filters were re-probed with an antibody directed against GAPDH. The results (Fig. 5) showed that proteins from several DENV isolates were not detected with the commercial antibodies used (which were mostly raised against DENV 2 proteins). However, clear reductions in protein expression as a consequence of orlistat pre-treatment were observed for the E protein of DENV 2LAB and ZIKV (Fig. 5), while JEV E protein levels were seen to be increased as a consequence of orlistat pre-treatment. Similarly, NS1 protein expression was reduced for DENV 2LAB and ZIKV, but increased for JEV. Consistent with previous results^27^ there was no apparent reduction of NS3 protein expression for DENV 2LAB, although there were reductions in expression of NS3 from DENV 4LAB, DENV 2DF and DENV 4DF.

## Discussion

Infections with mosquito transmitted viruses remain a significant public health burden worldwide. While human pathogenic viruses belong to a number of viral families, the most significant viruses in terms of public health belong to the genera *Alphavirus* and *Flavivirus*. Protective vaccination can offer a long-term solution to mosquito transmitted viruses, and effective, widely available vaccines are commercially available for JEV and YFV. However, these viruses remain the exception, and commercial vaccines for the other viruses in these two genera are either not yet available^36^ or of uncertain broad utility^37^. However, the number of viruses in question make it unlikely that vaccines will be developed for all the pathogenic viruses in these two genera, and the potential for the emergence of viruses not previously considered a significant pathogen of concern (as exemplified by the sudden emergence of ZIKV^11^) suggests that a reliance on vaccination alone is not a viable long term solution.

Similarly, drug development for mosquito transmitted viruses remains incomplete, and there is no commercially available drug to specifically treat any infection with viruses belonging to either the *Alphavirus* or *Flavivirus* genera. Again, development of specific drugs for each virus seems to be an unsustainable long-term policy for the same reasons as specific vaccines for each virus seems to be unsustainable. A viable alternative approach is the development of drugs that show broad utility against a number of viruses of either the same genera or multiple genera. Given the similarities between viruses of the genus *Alphavirus* and the genus *Flavivirus*, these are likely to be susceptible to manipulation of the same cellular pathways, despite distinct modes of replication.

A number of studies have implicated lipids and/or lipid metabolism in the replicative cycle of both flaviviruses and alphaviruses. Lipids are required at multiple stages of the replication cycle including but not limited to, virus entry, formation of the replication vesicles, genomic RNA encapsulation, virion formation and possibly virion release from the cell, and as such modulation of lipid metabolism is a good candidate for disruption of the virus replication cycle. In this study we investigated the effect of orlistat against a panel of RNA viruses, representative of DENVs of different origins (laboratory adapted, low passage from different disease severities) as well as JEV, ZIKV and CHIKV. The results showed a great deal of variation in the effect of orlistat on different viruses, particularly under different treatment regimens. Different levels of response to a drug have been previously reported for DENV. Milligan and colleagues reported markedly different EC50 values for a panel of DENVs evaluated for response to NITD-008^38^, while Talarico and colleagues showed serotype specific responses to sulfated polysaccharides^39^.

For DENV, a short-term pre-treatment of cells 1 h before infection resulted in a reduction in the number of released genomes for five of the nine DENV isolates tested. However for three of these isolates (DENV 1DHF, DENV 3DF and DENV 4DF), the reduced genome copy number was not reflected in a significant reduction in virus titer (albeit that virus titer was reduced for all three viruses, but not significantly). Indeed, virus titer was only reduced by around 1 log for six of the nine isolates tested, and the level of infection as assessed by flow cytometry was only reduced for five of the nine isolates. However, studies have shown that DENV severity is associated with the level of virus in the blood^40^, and thus orlistat treatment, even at low systemic levels could have some effect on the course of the diseases. For the other viruses tested with short-term pre-treatment, no significant change was observed in the level of infection for both isolates of ZIKV, although virus titer and genome copy number were reduced. For CHIKV, a significant reduction was seen in virus titer and genome copy number for both isolates examined, but only one of the two isolates was seen to have a reduction in the level of infection, albeit that the reduction was relatively small. JEV was the most discordant virus investigated, with an increase observed in the level of infection and viral protein expression, whilst both virus titer and genome copy number were reduced. These results would suggest that orlistat short term pre-treatment affects a number of processes, with these processes impacting differently on different viruses at different stages of the replication cycle. In particular for JEV it suggests that virus particle formation and egress are affected, possibly through insufficient membrane fluidity^16^, which would result in increased antigen inside the cell, and decreased virus and genome outside the cell, as seen here.

The longer term treatment exposures (post-treatment only, and pre- and post-infection treatments) also showed variation with respect to the different viruses. In some cases, namely DENV 4/163 for both treatment modalities, and ZIKV MR766 under post-treatment only, the level of infection was increased. Furthermore, the genome copy number in the culture supernatants was not significantly reduced for several viruses (See Table 1). Notably, virus titer was reduced for all viruses under both treatment regimens, in some cases by 3–4 log (Fig. 3). The discordant results between level of infection, genome copy number and virus titer seen under higher treatment levels again point towards orlistat affecting viral replication at several points of the replication cycle.

Orlistat is able to irreversibly inhibit the thioesterase domain of FASN through the action of a highly reactive beta-lactone group that covalently binds the active site serine residue (Ser 2308)^41^. Studies have additionally shown that the reactive beta-lactone group of orlistat can inhibit the activity of multiple cellular enzymes^42^. Other inhibitors of FASN such as cerulenin and C-75 have similar off target effects^43^. However, despite the lack of specificity of orlistat, the drug is clinically approved, and in some countries available without a prescription. These advantages may outweigh the disadvantages.

This study utilized a common concentration of 100 μM orlistat to determine the effects on the viruses. Although somewhat high, previous studies have evaluated the effect of other drugs at this concentration or higher, and subsequently gone on to show antiviral effects *in vivo*^44,45^.

Overall, orlistat was shown to have promising activity against a number of flaviviruses, albeit with less efficacy with a single short-term pre-treatment. A long-termer post-treatment as well as a combination of pre- and post-treatments caused a marked reduction in titer for all viruses investigated, and as it is believed that viral levels in the blood are associated with disease severity^40^, orlistat possibly has useful clinical applications. However, a significant question that remains to be addressed is whether clinically relevant levels of orlistat can be achieved in the course of the disease. The results showing discrepancy between infection level, protein expression and the titer and genome copies of released virus, suggest that orlistat has a predominant effect at several steps of the viral replication cycle. However, this study also clearly shows that the response of flaviviruses to orlistat treatment varied significantly, suggesting that an effective antiviral treatment may need to simultaneously target multiple cellular processes.

## Supplementary information

Supplementary info.

## Data Availability

All data generated or analysed during this study are included in this published article (and its Supplementary Information files).
