# Supplementary material for: Evaluation of the antiviral activity of orlistat (tetrahydrolipstatin) against dengue virus, Japanese encephalitis virus, Zika virus and chikungunya virus
Source: Sci Rep. 2020 Jan 30;10:1499. doi: 10.1038/s41598-020-58468-8 (PMC6992670; doi:10.1038/s41598-020-58468-8)

Supplemental Materials

Evaluation of the antiviral activity of orlistat (tetrahydrolipstatin) against dengue virus, Japanese encephalitis virus, Zika virus and chikungunya virus

Atitaya Hitakarun[1], Sarawut Khongwichit[1], Nitwara Wikan[1], Sittiruk Roytrakul[2], Sutee Yoksan[1], Supoth Rajakam[1], Andrew D. Davidson[3], and Duncan R. Smith[1]*

[1]Institute of Molecular Biosciences, Mahidol University, Salaya, 73170, Thailand
[2]National Center for Genetic Engineering and Biotechnology (BIOTEC), National Science and Technology Development Agency, Pathum Thani, 12120, Thailand
[3]School of Cellular and Molecular Medicine, University of Bristol, BS8 1TD, United Kingdom

*Correspondence to: Duncan R. Smith, Molecular Pathology Laboratory, Institute of Molecular Biosciences, Mahidol University, Salaya Campus, 25/25 Phuttamonthon Sai 4, Salaya, Nakhon Pathom, Thailand 73170; Phone: 66(0) 2441-9003 to 7. Fax: 66 (0) 2441-1013. E-mail: duncan_r_smith@hotmail.com, duncan.smi@mahidol.ac.th

Supplemental Figure 1. Determination of orlistat cytotoxicity to HEK293T/17 cells with different protocols
Supplemental Figure 2. HEK293T/17 cells morphology after orlistat pre-treatment
Supplemental Figure 3. HEK293T/17 cells morphology after orlistat post-treatment
Supplemental Figure 4. HEK293T/17 cells morphology after orlistat pre- and post-treatment
Supplemental Figure 5. Evaluation of DENV infection of HEK293T/17 cells
Supplemental Figure 6. Non-normalized infection data for ZIKV, JEV and CHIKV
Supplemental Figure 7. Effect of orlistat pre-treatment on JEV infection and titer
Supplemental Table 1. Summary of viruses used
Supplemental Table 2. Specific primers sequences used for qRT-PCR
Supplemental references
Uncropped western blots

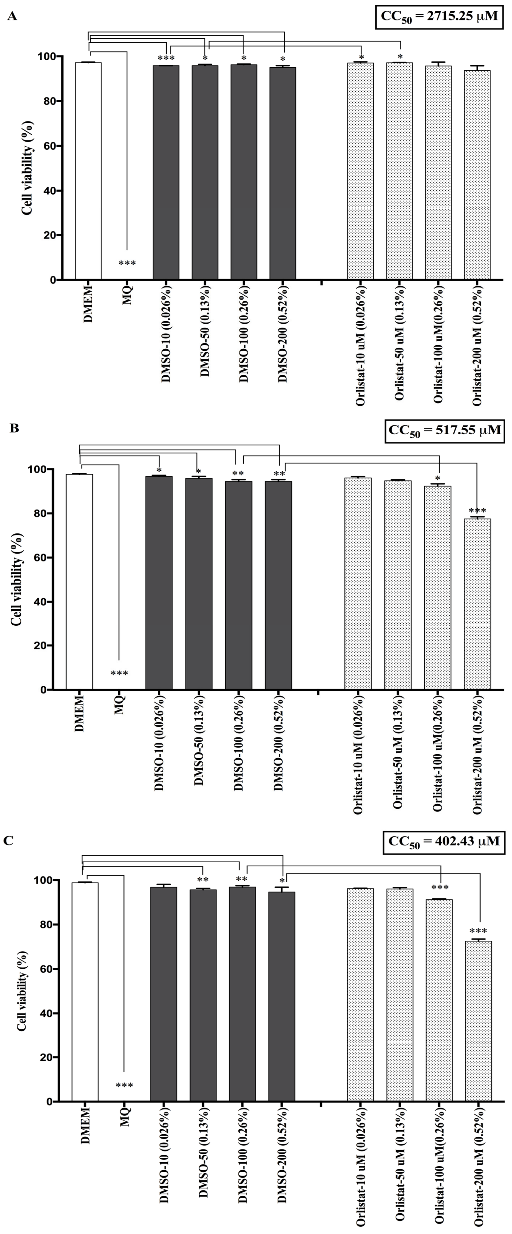

Supplemental Figure 1. Determination of orlistat cytotoxicity to HEK293T/17 cells. HEK293T/17 cells were incubated with different concentrations of orlistat, or DMSO vehicle control (v/v) for (A) 1 h pre-treatment, (B) post-treatment, or (C) pre- and post-combined treatment for 36 hours followed by a Trypan Blue viability test. Treatment with milli-Q water was used as a positive control for non-viable cells and DMEM for viable cells. All experiments were undertaken independently in triplicate with quadruplicate counting. Bars show mean +/- SD (*; p value <0.05)

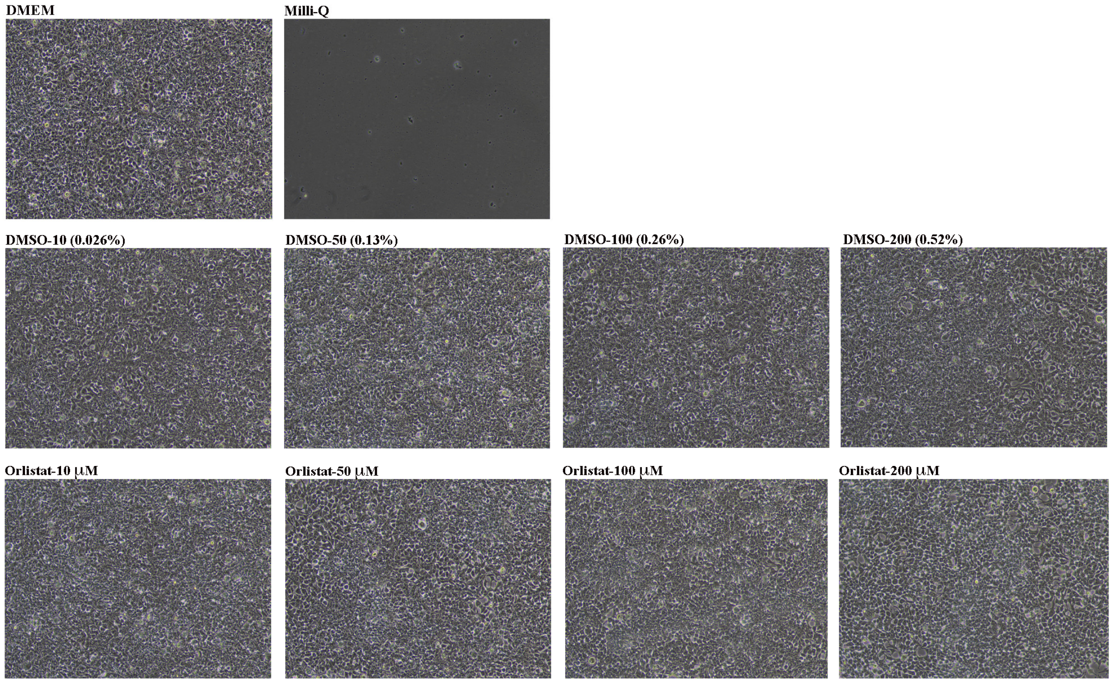

Supplemental Figure 2. HEK293T/17 cell morphology after orlistat pre-treatment. HEK293T/17 cells were pre-treated with different concentrations of orlistat, DMSO vehicle control, milli-Q water, or DMEM and incubated for 36 h followed by observation under an inverted microscope. Magnification x 20.

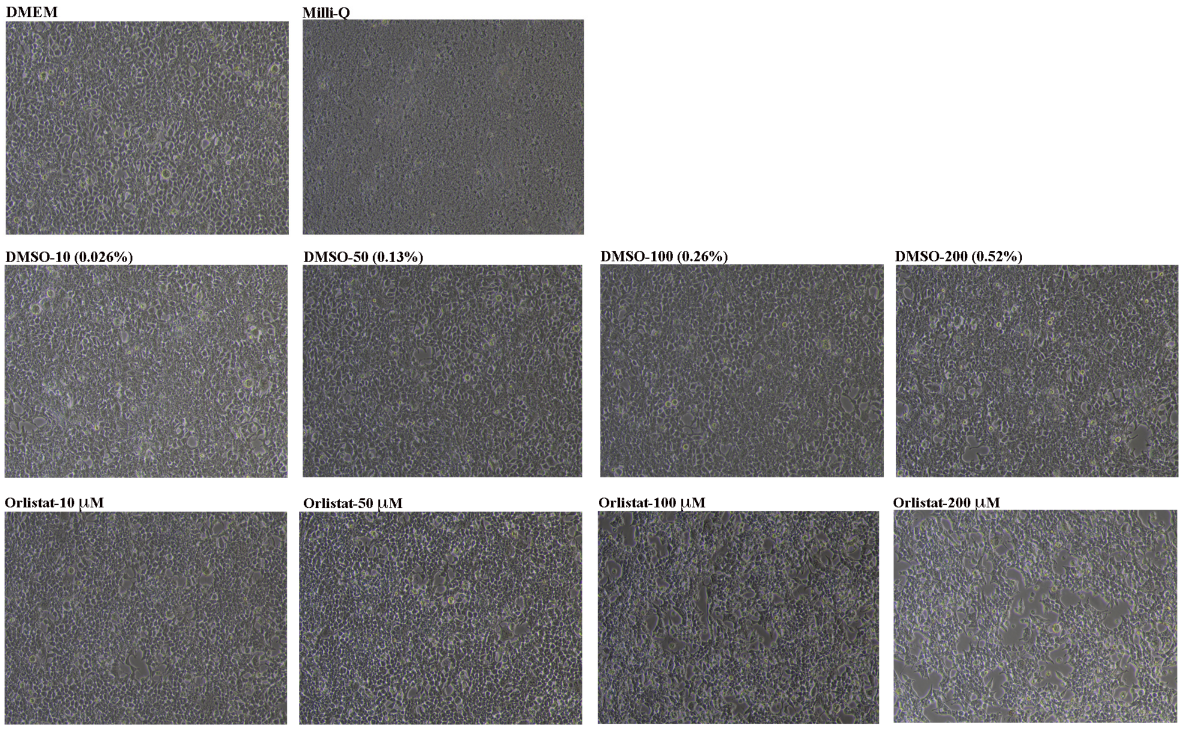

Supplemental Figure 3. HEK293T/17 cell morphology after orlistat post-treatment. HEK293T/17 cells were incubated with different concentrations of orlistat, DMSO vehicle control, milli-Q water, or DMEM for 36 h followed by observation under an inverted microscope. Magnification x 20.

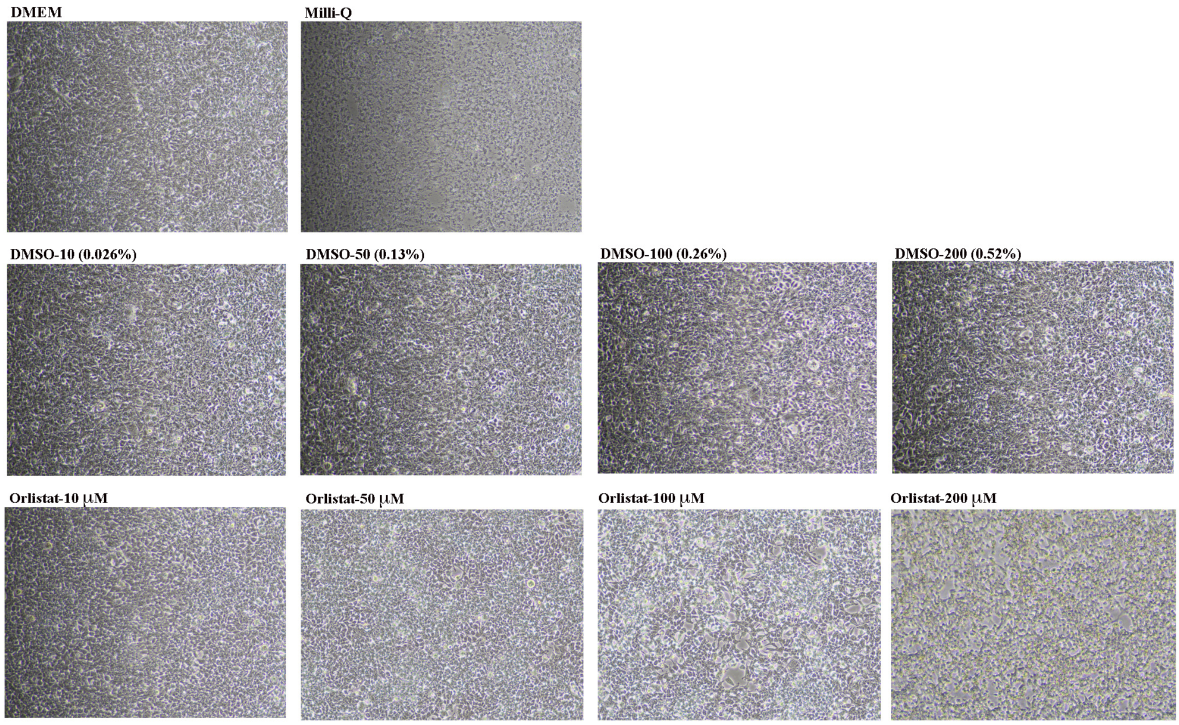

Supplemental Figure 4. HEK293T/17 cell morphology after orlistat pre- and post-treatment.
HEK293T/17 cells were pre-treated with different concentrations of orlistat, DMSO vehicle control, milli-Q water, or DMEM and incubated for 36 h in the presence of orlistat followed by observation under an inverted microscope. Magnification x 20.

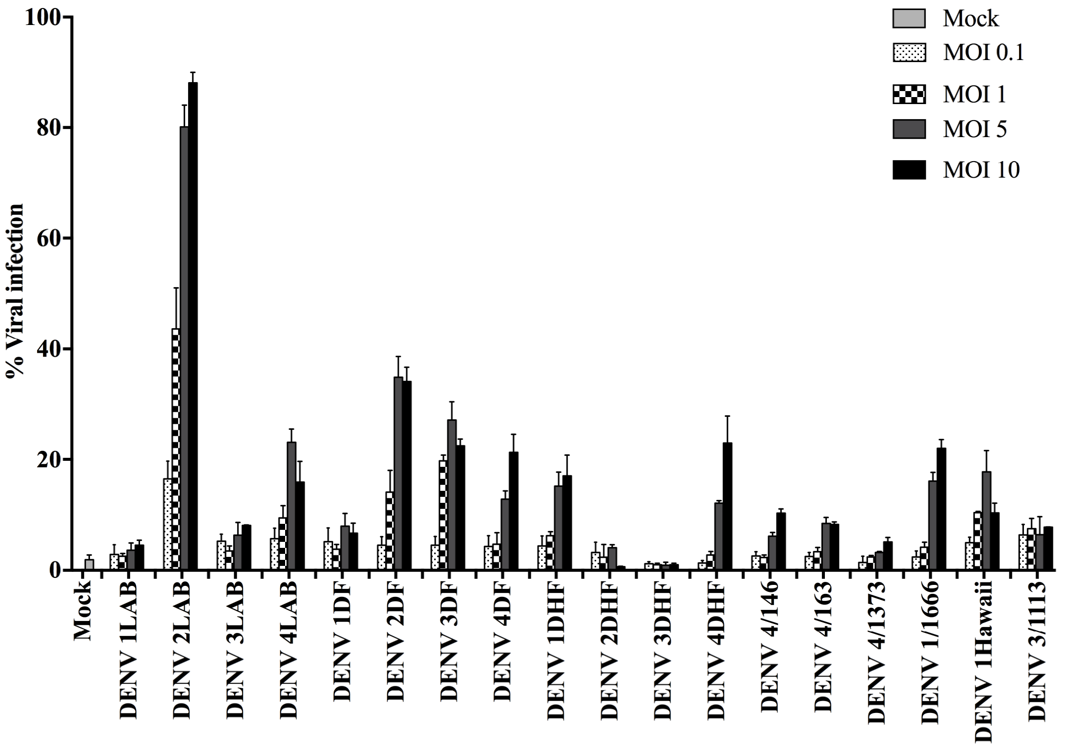

Supplemental Figure 5. Evaluation of DENV infection of HEK293T/17 cells. HEK293/17 cells were mock infected or infected with one of 18 DENV isolates namely DENV 1LAB, DENV 1- Hawaii, DENV 2LAB, DENV 3LAB, DENV 4LAB, DENV 1DF, DENV 2DF, DENV 3DF, DENV 4DF, DENV 1DHF, DENV 2DHF, DENV 3DHF, DENV 4DHF, DENV 1/1666, DENV 3/1113, DENV 4/146, DENV 4/163, and DENV 4/1373 at MOIs ranging from 0.1 to 10 and level of infection was determined at 24 h.p.i by flow cytometry. All experiments were undertaken independently in triplicate.

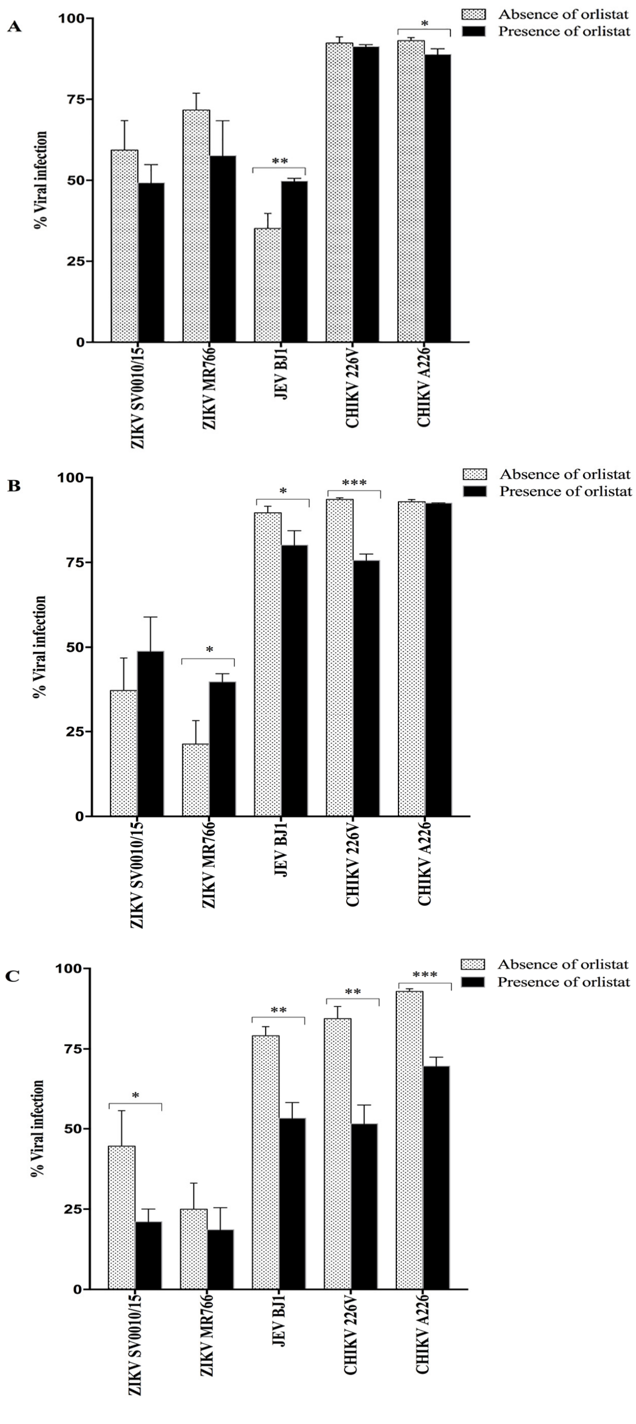

Supplemental Figure 6. Non-normalized infection data for ZIKV, JEV and CHIKV. Data of different treatment protocols (A) pre-treatment, (B) post-treatment, or (C) pre- and post-combined treatment are plotted as normalized in Figure 2B, 2D, and 2F, respectively. Bar graphs show mean +/- SD (*;p value < 0.05, **; p value < 0.01, and ***; p value < 0.001).

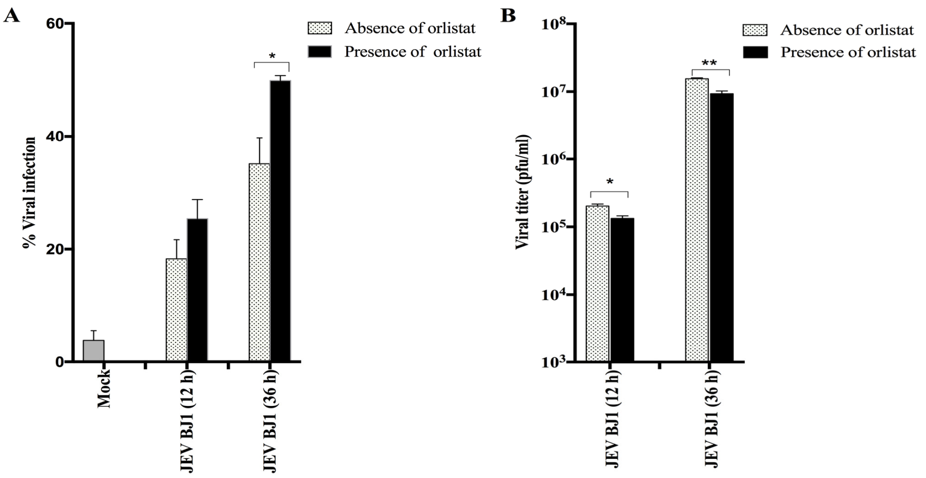

Supplemental Figure 7. Effect of orlistat pre-treatment on JEV infection and titer. HEK293T/17 cells were pre-treated with a vehicle control of DMSO or 100 µM orlistat and were subsequently infected at MOI of 5 with one isolate of JEV (BJ1), At 12 and 36 h.p.i. viral (A) level of infection was determined by flow cytometry, and (B) virus titers in the supernatants were determined by standard plaque assay. Experiments were undertaken independently in triplicate with duplicate plaque assay. Bar graphs show mean +/- SD (*;p value < 0.05, **; p value < 0.01, and ***; p value < 0.001).

Supplemental Table 1. Summary of viruses used.

Serotype	Strain	Designation	Passage history	Reference	GenBank accession number
DENV-1	16007	DENV 1LAB	High Passage	[Huang et al., 2000]	AF180817
	SS12/61	DENV 1DF	Low Passage	[Rungruengphol et al., 2015]	KM519585
	SS12/60	DENV 1DHF	Low Passage	[Rungruengphol et al., 2015]	KM519584
	Hawaii	DENV 1Hawaii	High Passage	[Anez et al., 2016]	KM204119.1
	SS11/1666	DENV 1/1666	Low Passage	This study	MK301277
DENV-2	16681	DENV 2LAB	High Passage	[Blok et al., 1989]	M84727
	SS12/62	DENV 2DF	Low Passage	[Rungruengphol et al., 2015]	KM519586
	SS12/63	DENV 2DHF	Low Passage	[Rungruengphol et al., 2015]	KM519587
DENV-3	16562	DENV 3LAB	High Passage	[Rungruengphol et al., 2015]	KM519588
	SS12/64	DENV 3DF	Low Passage	[Rungruengphol et al., 2015]	-[a]
	SS12/65	DENV 3DHF	Low Passage	[Rungruengphol et al., 2015]	KM519589
	SS15/1113	DENV 3/1113	Low Passage	This study	MK301278
DENV-4	1036	DENV 4LAB	High Passage	[Rungruengphol et al., 2015]	KM519590
	SS12/66	DENV4DF	Low Passage	[Rungruengphol et al., 2015]	KM519591
	SS12/67	DENV 4DHF	Low Passage	[Rungruengphol et al., 2015]	KM519592
	SS14/146	DENV 4/146	Low Passage	This study	MK301274
	SS14/163	DENV 4/163	Low Passage	This study	MK301275
	SS11/1373	DENV 4/1373	Low Passage	This study	MK301276
ZIKV	SV0010/15	ZIKV SV0010/15	Low Passage	[Ellison et al., 2016]	KX051562
	MR766	ZIKV MR766	High Passage	Unpublished	MK105975
JEV	BJ1	JEV BJ1	High Passage	[Hashimoto et al., 1988]	L48961
CHIKV	ECSA E1: 226 V	CHIKV 226V	Low Passage	[Wikan et al., 2012]	-[b]
	ECSA E1:A226	CHIKV A226	Low Passage	[Wikan et al., 2012]	-[b]

[a] No GenBank accession number is available (as reported in [Rungruengphol C., et al., 2015]).

[b] No GenBank accession numbers available. Viruses as reported in [Wikan N., et al., 2012]).

Supplemental Table 2. Specific primers sequences used for qRT-PCR.

Viruses	Genes	Primer sequence (Forward and reverse)	Reference
DENV	*NS1*	5'-TGC TGA CAT GGG TTA TTG GAT AG-3'<br>5'-ACT CCA TTG CTC CAC AGT GTG TG-3'	[Panraksa et al., 2017]
ZIKV	*E*	5'-TTG GAG GAA TGT CCT GGT TCT CAC-3'<br>5'-AGT CAG GAT GGT ACT TGT ACC-3'	KX051562.1
JEV	*NS3*	5'-AGA GCG GGG AAA AAG GTC AT-3'<br>5'-TTT CAC GCT CTT TCT ACA GT-3'	[Santhosh et al., 2007]
CHIKV	*nsP2*	5'-GCA CGT CAA CGT ACT CCT AAC-3'<br>5'-GGG TTC TGC AGC GTC TTT AT-3'	[Khongwichit et al., 2016]

Supplemental references

Anez G, Heisey DA, Volkova E, Rios M. (2016). Complete Genome Sequences of Dengue Virus Type 1 to 4 Strains Used for the Development of CBER/FDA RNA Reference Reagents and WHO International Standard Candidates for Nucleic Acid Testing. Genome Announc, 4.

Blok J, Samuel S, Gibbs AJ, Vitarana UT. (1989). Variation of the nucleotide and encoded amino acid sequences of the envelope gene from eight dengue-2 viruses. Arch Virol, 105:39-53.

Ellison DW, Ladner JT, Buathong R, Alera MT, Wiley MR, Hermann L, Rutvisuttinunt W, Klungthong C, Chinnawirotpisan P, Manasatienkij W, Melendrez MC, Maljkovic Berry I, Thaisomboonsuk B, Ong-Ajchaowlerd P, Kaneechit W, Velasco JM, Tac-An IA, Villa D, Lago CB, Roque VG, Jr., Plipat T, Nisalak A, Srikiatkhachorn A, Fernandez S, Yoon IK, Haddow AD, Palacios GF, Jarman RG, Macareo LR. (2016). Complete Genome Sequences of Zika Virus Strains Isolated from the Blood of Patients in Thailand in 2014 and the Philippines in 2012. Genome Announc, 4.

Hashimoto H, Nomoto A, Watanabe K, Mori T, Takezawa T, Aizawa C, Takegami T, Hiramatsu K. (1988). Molecular cloning and complete nucleotide sequence of the genome of Japanese encephalitis virus Beijing-1 strain. Virus Genes, 1:305-317.

Huang CY, Butrapet S, Pierro DJ, Chang GJ, Hunt AR, Bhamarapravati N, Gubler DJ, Kinney RM. (2000). Chimeric dengue type 2 (vaccine strain PDK-53)/dengue type 1 virus as a potential candidate dengue type 1 virus vaccine. J Virol, 74:3020-3028.

Khongwichit S, Wikan N, Abere B, Thepparit C, Kuadkitkan A, Ubol S, Smith DR. (2016). Cell-type specific variation in the induction of ER stress and downstream events in chikungunya virus infection. Microb Pathog, 101:104-118.

Panraksa P, Ramphan S, Khongwichit S, Smith DR. (2017). Activity of andrographolide against dengue virus. Antiviral Res, 139:69-78.

Rungruengphol C, Jaresitthikunchai J, Wikan N, Phaonakrop N, Keadsanti S, Yoksan S, Roytrakul S, Smith DR. (2015). Evidence of plasticity in the dengue virus: Host cell interaction. Microb Pathog, 86:18-25.

Santhosh SR, Parida MM, Dash PK, Pateriya A, Pattnaik B, Pradhan HK, Tripathi NK, Ambuj S, Gupta N, Saxena P, Lakshmana Rao PV. (2007). Development and evaluation of SYBR Green I-based one-step real-time RT-PCR assay for detection and quantitation of Japanese encephalitis virus. J Virol Methods, 143:73-80.

Wikan N, Sakoonwatanyoo P, Ubol S, Yoksan S, Smith DR. (2012). Chikungunya virus infection of cell lines: analysis of the East, central and South african lineage. PLoS One, 7:e31102.

Supplemental Materials: Uncropped western blots

**Evaluation of the antiviral activity of orlistat (tetrahydrolipstatin) a
gainst dengue virus, Japanese encephalitis virus, Zika virus and chikungunya virus**

Atitaya Hitakarun[1], Sarawut Khongwichit[1], Nitwara Wikan[1], Sittiruk Roytrakul[2], Sutee Yoksan[1],
Supoth Rajakam[1], Andrew D. Davidson[3], and Duncan R. Smith[1]*

[1]Institute of Molecular Biosciences, Mahidol University, Salaya, 73170, Thailand

[2]National Center for Genetic Engineering and Biotechnology (BIOTEC), National Science and Technology Development Agency,
Pathum Thani, 12120, Thailand
[3]School of Cellular and Molecular Medicine, University of Bristol, BS8 1TD, United Kingdom

*Correspondence to: Duncan R. Smith, Molecular Pathology Laboratory, Institute of Molecular Biosciences,
Mahidol University, Salaya Campus, 25/25 Phuttamonthon Sai 4, Salaya, Nakhon Pathom, Thailand 73170;
Phone: 66(0) 2441-9003 to 7. Fax: 66 (0) 2441-1013. E-mail: duncan_r_smith@hotmail.com, duncan.smi@mahidol.ac.th

E-protein

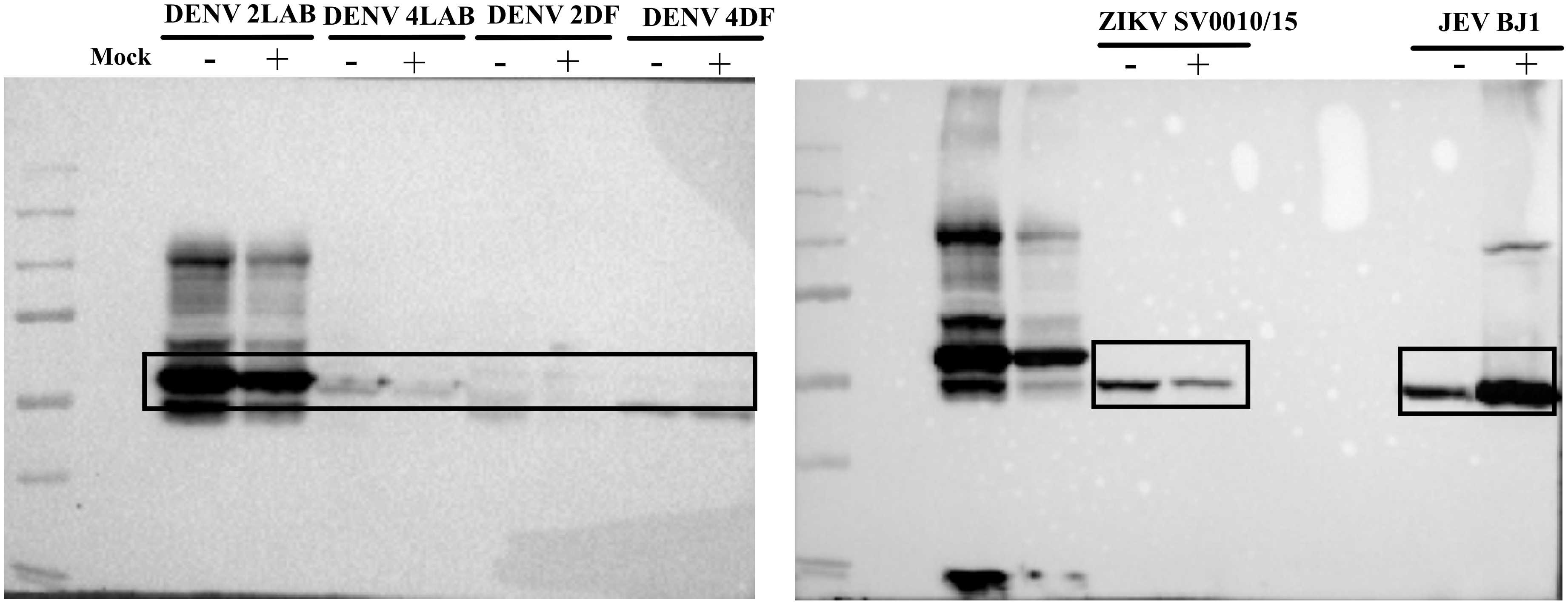

DENV 2LAB
DENV 4LAB
DENV 2DF
DENV 4DF
Mock
- + - + - + - +
ZIKV SV0010/15
JEV BJ1
- + - +

NS1-protein

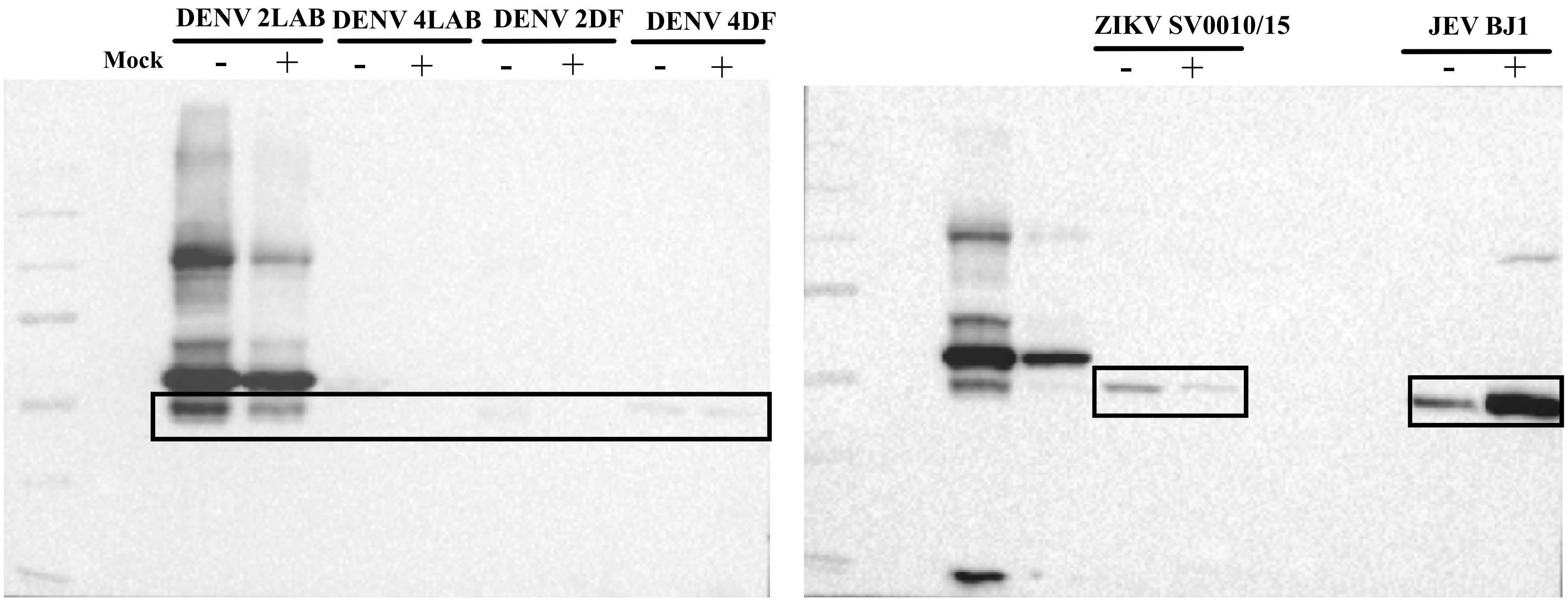
DENV 2LAB
DENV 4LAB
DENV 2DF
DENV 4DF
Mock
- + - + - + - +
ZIKV SV0010/15
JEV BJ1
- + - +

NS3-protein

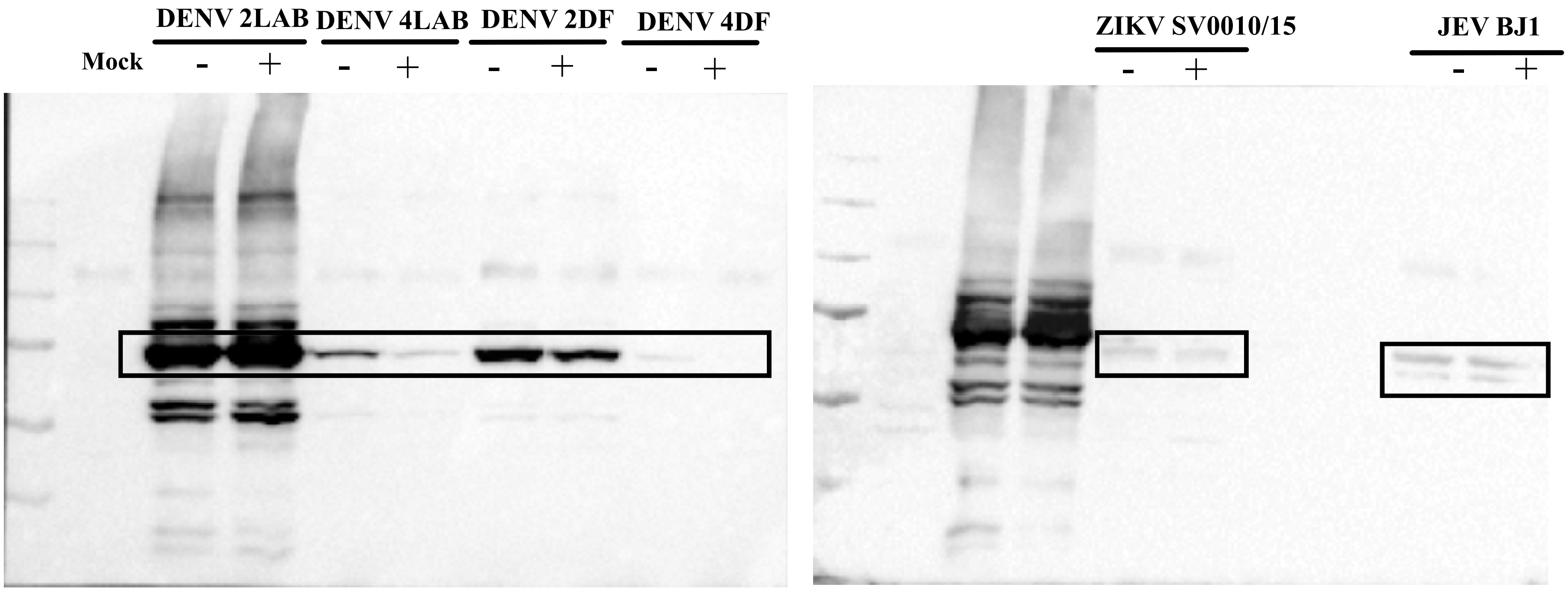
DENV 2LAB
DENV 4LAB
DENV 2DF
DENV 4DF
Mock
- + - + - + - +
ZIKV SV0010/15
- +
JEV BJ1
- +

NS5-protein

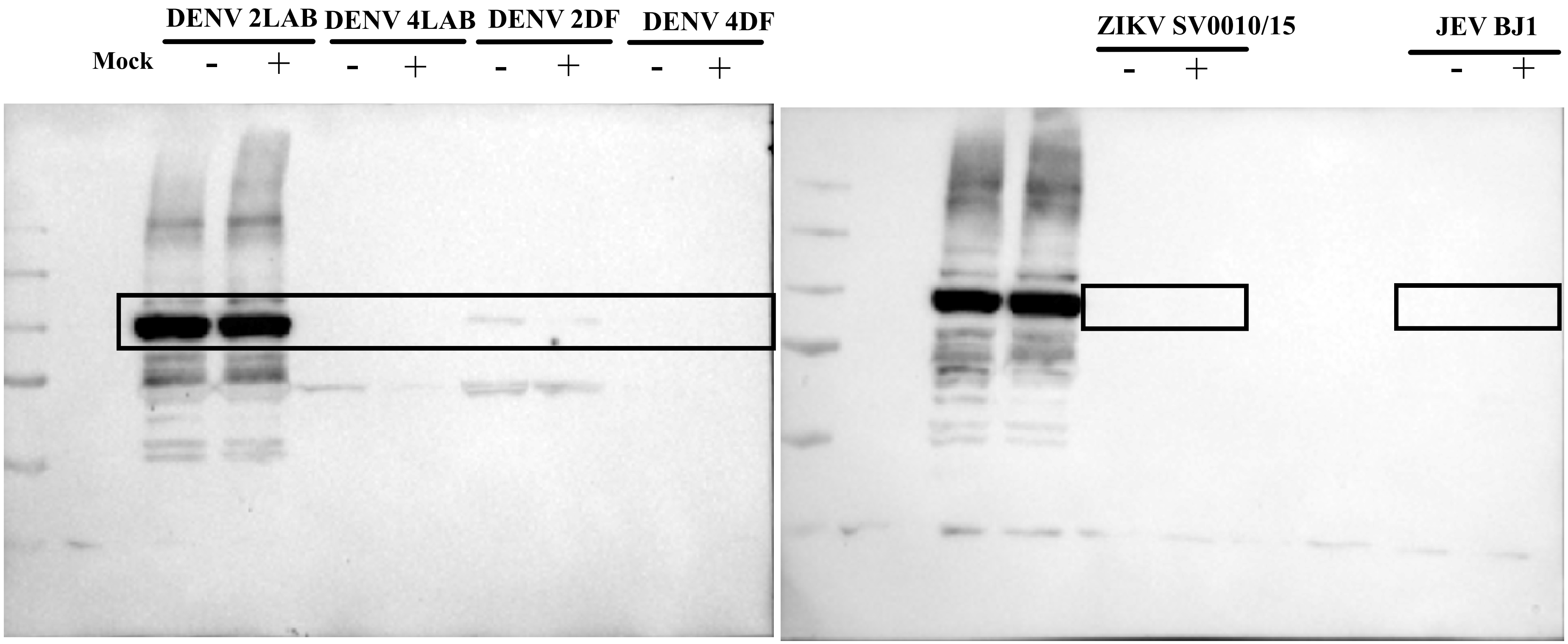

DENV 2LAB
DENV 4LAB
DENV 2DF
DENV 4DF
Mock
- +
- +
- +
- +
ZIKV SV0010/15
- +
JEV BJ1
- +

GADPH-protein

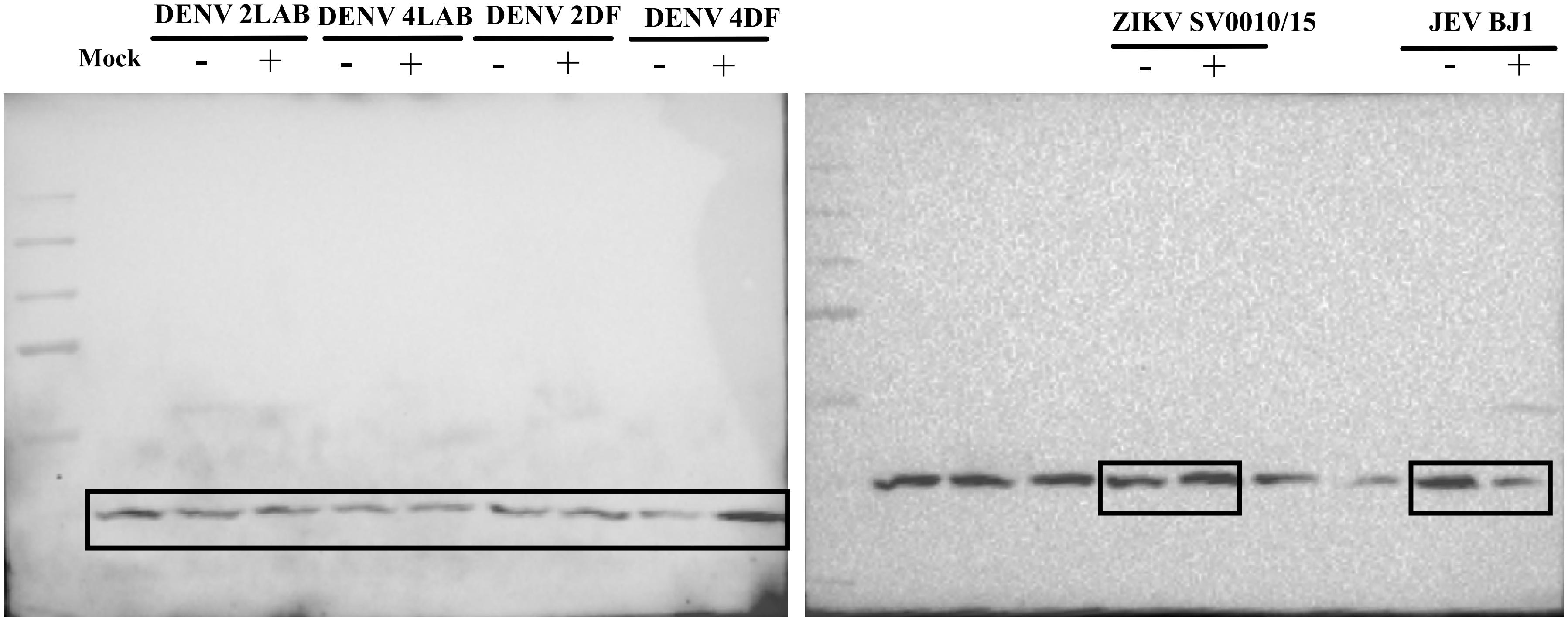

Supplement: Supplementary file 1 — Supplementary info. [file 41598_2020_58468_MOESM1_ESM.pdf]
